# Efficient treatment of esophageal nutrition bezoars: dissolution outmatches removal—the Zurich approach

**DOI:** 10.1007/s12328-021-01516-1

**Published:** 2021-09-09

**Authors:** Bernhard Morell, Philipp Karl Buehler, Patrick Raphael Bader, Silvia Lang, Michael Scharl, Christoph Gubler, Fritz Ruprecht Murray

**Affiliations:** 1 Division of Gastroenterology and Hepatology, University Hospital Zurich, Rämistrasse 100, 8091 Zurich, Switzerland; 2 Institute of Intensive Care, University Hospital Zurich, Rämistrasse 100, 8091 Zurich, Switzerland

**Keywords:** Enteral feed bezoar, Sodium bicarbonate, Enteral feeding

## Abstract

Enteral feed bezoars are difficult to treat and can lead to serious adverse events. There is no standardized treatment approach and various strategies have been suggested. We herein describe three cases of successful dissolutions of feed bezoars consisting of Promote^®^ Fibre Plus with sodium bicarbonate 8.4% in critically ill patients. To provide the rationale for this approach, the effect of sodium bicarbonate 8.4% on enteral feed concretions was studied in vitro. First, Promote^®^ Fibres Plus was incubated with hydrochloric acid with gradually decreasing pH values to establish a pH at which the solution solidifies. The resulting enteral feed concretion was exposed to sodium bicarbonate 8.4% and Coca Cola^®^. All patients were successfully treated with sodium bicarbonate 8.4% without the need of lengthy or repeat endoscopies. In vitro, Promote^®^ Fibres Plus solidifies when acidified below a pH of 4.6. The resulting enteral feed concretions dissolved when exposed to sodium bicarbonate 8.4%. Incubation with Coca Cola^®^ had no effect. We provide evidence that enteral feed bezoars consisting of Promote^®^ Fibres Plus can be efficiently and safely treated with sodium bicarbonate 8.4% offering a new approach for daily patient care.

**Supplementary Information:**

The online version contains supplementary material available at 10.1007/s12328-021-01516-1.

## Introduction

Bezoars are retained collections of indigestible foreign material in the gastrointestinal tract that most commonly accumulate in the stomach [1, 2]. Rarely, bezoars originate in the esophagus, causing esophageal obstruction. Bezoars have been traditionally categorized according their components: phytobezoars, trichobezoars, lactobezoar, and pharmacobezoar [2]. Enteral feed bezoars, however, may be regarded as an uncommon complication of enteral feeding. In a retrospective study among 1003 ICU patients receiving enteral nutrition, 9 (0.9%) developed esophageal impaction due to enteric nutrition [3]. The formation of enteral feed bezoars is incompletely understood. Limited evidence suggests that the formation of enteral feed bezoars is triggered by gastro-esophageal reflux since acidic pH causes casein as component of the enteric formula to solidify [4]. In line, a comprehensive literature review demonstrated that casein was part of all formulas that were implicated in the development of enteral feed bezoars [5]. On the other hand there is evidence that formulas with skim milk as protein component do not clot when exposed to acidic pH [4, 5]. Treatment of enteral feed bezoars is challenging and no standardized approach to this clinical problem exists. Endoscopic removal of an esophageal bezoar may require several long lasting sessions [5] and perforation as consequence of endoscopic removal has been described [4]. Thus, pharmacologic treatment to dissolve the bezoars appears to be a reasonable alternative. Effervescent liquids (Coca Cola) [6] to soften the mass as well as dissolution of the mass using pancreatic enzyme extract [7] or N-Acetylcysteine [8] have been suggested. Lastly, to the best of our knowledge, there is only one case report that describes the successful use of sodium bicarbonate (NaHCO_3_) through the working channel of the endoscope to enable endoscopic removal of a massive enteral feed bezoar [9].

Herein, we describe the successful and minimally invasive treatment of four cases presenting with large esophageal enteral feed bezoars using NaHCO_3_, administered through a nasogastric tube (NGT). In addition to that, we investigate whether the effect of NaHCO_3_ can be replicated in vitro by exposing an artificial enteral feed bezoar to NaHCO_3_.

### Case reports

Following an initial case (for a detailed description please find the “index patient” in the supplementary file) of an enteral feed bezoar with an unsuccessful endoscopic extraction attempt and first evidence of bezoar dissolution with NaHCO_3_ 8.4% (= 8.4 g per 100 ml), three other cases of enteral feed bezoars were successfully treated with slow continuous drip line infusion of NaHCO_3_ via an endoscopically placed NGT just above the bezoar. In response to the information gathered analyzing the index patient, the treatment strategy was adjusted. In patients 1–3, continuous NaHCO_3_ 8.4% drip was administered slowly over 48 h, resulting in complete bezoar dissolution in all cases. All patients were hospitalized in the intensive care unit (ICU) of the University Hospital Zurich, Zurich, Switzerland, in response to life-threatening medical conditions between April 2019 and May 2021. All patients were enteral fed during ventilation with Promote^®^ Fibres Plus [PFP], (Abbott AG Baar Switzerland) based on ESPEN Nutrition Guidelines [10]. The amount of sodium hydrogen carbonate administered had no clinical significant effect on metabolic disorders.

**Fig. 1:**
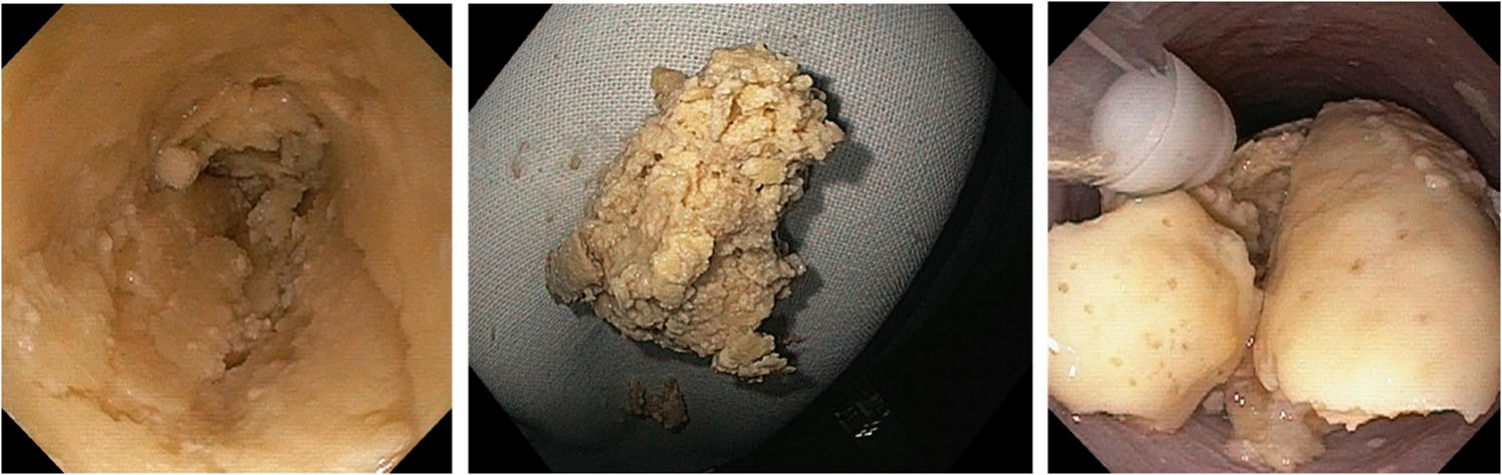
Representative endoscopic images of solidified enteric nutrition. **A** Completely occluded esophageal lumen. **B** Piece of a bezoar after incomplete extraction attempted with a net. **C** Endoscopic placement of a nasogastric tube (11 o’clock) just proximal to the bezoar

**Fig. 2:**
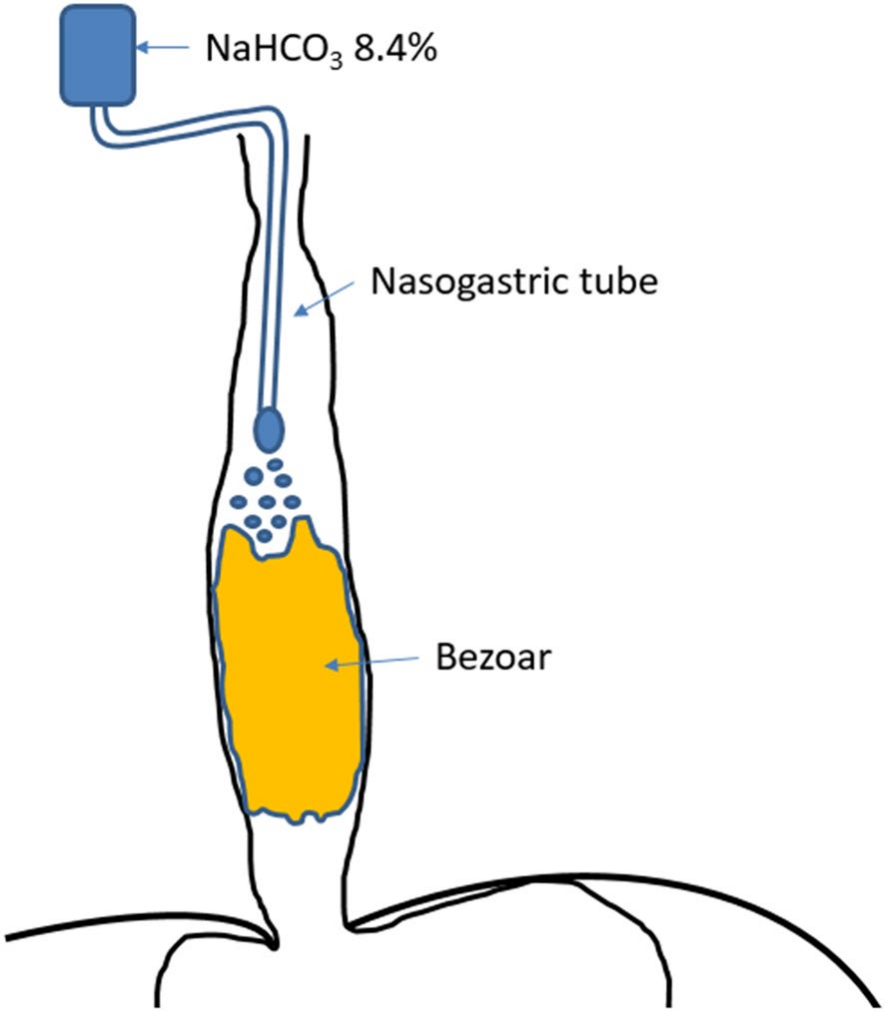
Schematic illustration of nasogastric tube location after endoscopic placement just proximal to the bezoar with continuous drip administration of NaHCO_3_ 8.4%

Table 1 summarizes patient characteristics. Figure 1 includes original endoscopic documentation of bezoars and Fig. 2 represents a schematic illustration of the therapeutic approach. All patients gave their informed consent prior to the inclusion in this manuscript.

**Table 1:** Case specific characteristics

Patient	Sex/age (years)	Reason for ICU admission/enteral nutrition	Type enteral nutrition	Acid suppressive therapy prior to bezoar?	Therapeutic approach bezoar	Bezoar dissolution successful? Duration of dissolution process (hours)	Sequelae	Proposed reason for bezoar
1	Male/69	Hypoxic shock	PFP	Yes	NaBic 8.4%	Yes/48	4 days of prolonged mechanical ventilation	Dislocation of NGT following vomiting
2	Female/59	Burn-injury covering 33% body surface area	PFP	No	NaBic 8.4%	Yes/48	4 days of prolonged mechanical ventilation	Dislocation of NGT
3	Male/80	Cerebrovascular accident following occlusion of internal carotid artery	PFP	Yes	NaBic 8.4%	Yes/48	Impossibility to administer clopidogrel	Dislocation of NGT following coughing

*PFP* Promote^®^ Fibres Plus, *NGT* nasogastric tube

To systematically confirm the hypothesis that PFP (pH 6.4) solidifies in an acidic environment and dissolves when exposed to NaHCO_3_ (representing an alkaline dissolvent) an in vitro experiment was conducted. The decision to use NaHCO_3_ as dissolvent was made in response to the availability and safety of its use.

After verification of the above mentioned hypothesis, the experiment was repeated with the second most often used enteral feed solution in our hospital, Nepro HP^®^ (Abbott AG Baar Switzerland), which showed the same characteristics (solidification and dissolution) as PFP.

Please find a description and representative images in the online resource as a supplementary file.

### Case descriptions

Patient 1 (P1), 69-year-old male, hospitalized after out-of-hospital-reanimation following pulmonal bleeding and consecutive cardiac arrest due to hypoxia. The patient’s medical history was remarkable for stage III COPD as well as s/p roux-en-y bypass following duodenal ulcer bleeding 4 years previously. A NGT was placed at admission and enteral feeding with PFP was started on hospitalization day 2. Within the following 6 days, PFP was gradually increased to a dose from 10 to 25 kcal/kg/bw/day. On day 9, recurrent vomiting occurred, resulting in a decrease of PFP dose and temporary start of parenteral nutrition. On day 11, the distal end of the NGT was assumed to be localized in the distal esophagus on chest X-ray. On the evening of the same day, the NGT was thought to be clogged. A gastroscopy on day 12, for which the patient had to be re-intubated, verified solidified feeding solution with blockage of the esophageal lumen as well as a dislocation of the NGT. After placing a new NGT with the tip just proximal the bezoar, dissolution with Coca Cola and ascorbic acid failed over the following 2 days. Starting on day 14, 20–30 ml boli of NaHCO_3_ 8.4% were repeatedly (every 3–4 h) administered via the NGT. Follow-up gastroscopy on day 16 finally verified a complete dissolution of the bezoar without remnants in the esophagus, allowing the placement of a duodenal feeding tube as well as extubation of the patient.

Patient 2 (P2), 59-year-old female, hospitalized following an accidental burn-injury covering 33% of her body surface area. Enteral feeding with PFP was started at admission (day 1) and gradually increased to a dose of 25 kcal/kg/bw/day. On day 7, the NGT was found to be clogged. A gastroscopy on the same day verified a dislocated NGT, solidified feeding solution, and complete blockage of the esophagus. Just as described in P1, a new NGT was placed with the tip just proximal of the bezoar. Due to experience of P1 and the index patient, continuous drip of NaHCO_3_ 8.4% (2.5 ml per hour) was administered intending to dissolve the bezoar. Analogous to P1, after 48 h of continuous drip administration the NGT was easily movable into the stomach. A follow-up gastroscopy on day 11 verified a completely dissolved bezoar without remnants in the esophagus, allowing the placement of a duodenal feeding tube. Of note, initially an extubation of the patient was planned on day 7, which, however, had to be postponed to after the dissolution of the bezoar on day 10.

Patient 3 (P3), 80-year-old male, hospitalized following an occlusion of the right internal carotid artery with consecutive ischemic stroke. Enteral feeding with PFP was started on day 3 with a gradual dose increased to 25 kcal/kg/bw/day. On day 6, the patient coughed throughout the day, probably resulting in a dislocation of the lying NGT. On day 7, the application of enteral drugs failed, leading to a removal of the NGT, which was clogged with PFP solution. A gastroscopy on the same day verified a complete blockage of the esophagus by solidified feeding solution. As described in P1 and 2, a NGT was endoscopically placed with the tip just above the bezoar and continuous NaHCO_3_ 8.4% drip (3.5 ml per hour) was started. Along the lines of the previously described cases, the NGT could easily be pushed into the stomach after 48 h of continuous NaHCO_3_ 8.4% treatment. Of note, extubation was not deferred, but antiplatelet therapy with clopidogrel, urgently needed after stenting of the internal carotid artery, could not be administered for 2 days.

## Discussion

The treatment of enteral feeding bezoars represents a considerable clinical challenge. In our view, repeated endoscopic attempts to disintegrate large esophageal bezoars should be avoided, since prolonged endoscopic sessions may cause considerable stress to critically ill patients and carry an intrinsic risk of procedure-related complications. Therefore, efficient dissolution of large enteral feed bezoars represents a more elegant and less invasive approach. In this case series, we demonstrate the feasibility and effectiveness of NaHCO_3_ 8.4% for the treatment of large esophageal bezoars. Additionally, we were able to demonstrate that artificially generated enteral feed bezoars can be easily dissolved in vitro using sodium bicarbonate whereas the administration of Coca Cola^®^ had no effect. We did not use pancreatic enzymes in our experiments because enzymatic extracts require sodium bicarbonate to be effective. Moreover, the results of our experiment imply that no additional effect can be expected from using pancreatic enzymes. Lastly, our experiments confirm that acid exposure promotes enteral feed bezoar formation.

The cause of bezoar formation is incompletely understood. The administration of either sucralfate or aluminum hydroxide are well known triggers of bezoar formation [5, 11]. Notwithstanding, the formation of enteral feed bezoars may occur in the absence of these medications as demonstrated in our case series. Since enteral formulas containing casein rapidly solidify when acidified to a pH below 5 in vitro [4] gastro-esophageal reflux has been suspected to promote esophageal bezoar formation*.* However, the exact role of acidification in vivo is largely unknown since only case reports or case series have evaluated potential mechanism of enteral feed bezoar formation. Interestingly, to the best of our knowledge there is no case report describing enteral feed bezoar formation when enteral feeding is administered via percutaneous gastrostomy.

Enteral feed bezoars are rare [3]. In the here described period (April 2019 and May 2021) approximately 4000 ICU patients were enteral fed in our hospital. Four of them (0.1%) developed clinically relevant bezoars. However, thinking globally, the unreported or even unrecognized absolute number of cases might be high. Thus, clinicians should be aware of this complication and implement measures for its prevention. Reducing both gastro-esophageal reflux and stasis by elevating the patient’s head appears to be reasonable. In addition to that, in patients with gastroparesis not responding to prokinetic agents and/or very high risk of aspiration postpyloric feeding is preferable [10]. The role of proton-pump inhibitors (PPI) in the prevention of enteral feeding bezoars has not been studied and remains unclear. Moreover, esophageal 24 h pH-metry is hardly ever performed in critical care units which is why the degree of gastro-esophageal reflux in critically ill patients on or off PPI is poorly established. However, at dosages commonly used for stress ulcer prophylaxis [12], pH in the esophagus may still be < 5 [13].

Because malposition of the NGT may be regarded as a prerequisite for enteral feed bezoar, correct placement with conformation of correct position is recommended, either by radiography or using capnography [14]. On the other hand, auscultation may not be dependable since a tube inadvertently positioned in the esophagus can cause a sound alike to that of air pumped in the stomach [14, 15]. In addition to that, routinely flushing of the feeding tube and monitoring gastric reflux are additional measures that recognize clotting or malposition of the NGT. Lastly, whenever situations occur that might lead to NGT dislocation, e.g., vomiting, coughing or even in delirious patients, verification of the correct positioning should be considered.

There are limitations of this descriptive study that deserve consideration. Since the bezoar formation was in vivo observed exclusively after administration of PFP it is uncertain whether the administration of NaHCO_3_ 8.4% solution is only efficient in this context. However, in vitro we were able to show that bezoars of a different enteral feed solution (Nepro HP^®^) did not only clot in an acidic environment, but could also easily be dissolved with NaHCO_3_ 8.4%. Because calcium caseinate as component of PFP and Nepro HP^®^ represents a casein derivative, it appears to be plausible that the formation of enteral feed bezoars in our patients is casein dependent, in accordance with the study of Turner [4]. Ultimately, further investigations are needed to answer the question whether sodium bicarbonate can be efficiently used for enteral feed bezoars that arise after concretion of other nutrition formulas. In addition to that, owing the retrospective nature of this analysis there was no standardized protocol regarding the drip rate. Therefore, we do not know how much time is needed to completely dissolve an enteral feed bezoar using NaHCO_3_ 8.4% which most likely depends on the difficult to determine size of bezoar. Nonetheless, repeated endoscopic examinations to answer this question would not have been justified. Patients 2 and 3 (similar cases that were treated with the experience of the index patient and P1) were approached in a more standardized fashion leading to a proposed dripping rate of 2–4 ml per hour. Since the administration of NaHCO_3_ 8.4% via NGT appears to be safe and easy to apply we recommend this treatment in patients with enteral feed bezoars as first line approach before more straining treatments are considered. However, it needs to be considered that NaHCO_3_ 8.4% is administered by slow continuous drip with rates between 2–4 ml per hour over 48 h to prevent reflux (Fig. 2).

## Conclusion

In summary, sodium bicarbonate 8.4% solution represents an efficient and safe method to treat enteral feed bezoars that arise after accumulation of PFP in the esophagus. More studies would be of interest to confirm our observations.

## Supplementary Information

Below is the link to the electronic supplementary material.

Supplementary file1 (DOCX 3697 KB)

## Abbreviations

NaHCO_3_: Sodium bicarbonate
NGT: Nasogastric tube
PFP: Promote^®^ Fibres Plus
Min: Minutes
PPI: Proton-pump inhibitor
