# Supplementary material for: Efficient treatment of esophageal nutrition bezoars: dissolution outmatches removal—the Zurich approach
Source: Clin J Gastroenterol. 2021 Sep 9;14(6):1602–6. doi: 10.1007/s12328-021-01516-1 (PMC8557172; doi:10.1007/s12328-021-01516-1)
Supplement: Supplementary file 1 — Supplementary file1 (DOCX 3697 KB) [file 12328_2021_1516_MOESM1_ESM.docx]

**Efficient treatment of esophageal nutrition bezoars: dissolution outmatches removal – the Zurich approach**

**Online Resource**

**Index patient**

A 78 years old male patient, hospitalized following an ST-segment elevation infarction (STEMI) complicated by a cardiogenic shock with out-of-hospital reanimation. Prior to further management on the ICU, the patient underwent coronary angiography with revascularisation of the left anterior descending artery and placement of three coronary stents as well as a ventricular assist device. A NGT was placed at admission on the ICU. Enteral feeding with PFP was started on hospitalization day 2 with gradual increase of dose from 5-25kcal/kg/bw/d. Due to increasing reflux, PFP was changed to Nepro ® HP (reason: less volume for the equal amount of calories) on day 6. However, on day 7 the NGT could not be moved anymore (neither pushed down the stomach, nor pulled out). Gastroscopy on day 7 finally verified an impaction of the NGT in the esophagus by solidified enteral feeding solution. During gastroscopy, extraction of the bezoar with a net was unsuccessful. Instead, a new NGT with tip placed at the proximal end of the bezoar was introduced. Via this new NGT, NaHCO_3_ 8.4% was administered via drip. Follow-up gastroscopy the next day (day 8) showed a remaining bezoar, which, in turn to the previous day, was soft enough to remove parts via net and push remands gently into the stomach. Of note, antiplatelet therapy with ticagrelor could not be administered for 2 days.

**In vitro experimental analysis of solidification and dissolution characteristics of PFP – experimental setup**

As a first step, PFP was repeatedly titrated with hydrochloric acid (HCl) to a pH at which PFP solidifies. As a control, sodium chloride 0.9% was used. The PFP-HCl/NaCl solutions were then incubated at 37°C in separate wells for a period of 50 minutes (min), with photographic documentation after 5, 15 and 50min.

In a second step, solidified pieces of PFP were placed in two separated tubes, which were then filled with (A) NaHCO_3_ 8.4% (pH = 8.3) and (B) Coca-Cola ® (pH = 2.3). Photo documentation of the reaction were conducted immediately, at 5min, 30min and 3 hours.

**In vitro experimental analysis of solidification characteristics of PFP (step 1)**

In a first step, PFP was mixed with a specifically manufactured solution of hydrochloric acid (HCl) and sodium chlorid (NaCl) 0.9% with decreasing pH-values of 8.4, 6.3, 5, 4, 3 and 1.8 as well as controls with ddH20 and Coca-Cola ® to establish a pH, at which PFP solidifies. After mixture, pH-values of the solutions were documented (image 3). The solutions were then incubated at 37°C in separated wells for a period of 50 minutes (min), with behaviour documentation immediately, at 5min, 15min and 50min.

After adding pure HCL, PFP clotted immediately, making a pH measurement impossible. At a pH of 4.5 (well A1) the solution started to clot after 5min. All other wells did not show any reaction, with no clots after 50min incubation and removal of supernatant.

In summary, PFP at pH-values ≥4.6 did not show any texture change. At pH-values <4.6, PFP started to fall out over time: the lower the pH, the faster PFP clotted. Image 3 and table 2 illustrate the in vitro solidification experiment.

**In vitro experimental analysis of dissolution characteristics of PFP (step 2)**

Representative images of the in vitro setup are shown in image 4. Coca-Cola ® did not show any effect after an incubation of 3 hours. In contrast, adding NaHCO_3_ 8.4% resulted in an immediate formation of bubbles, with quick and complete dissolution of solidified PFP.


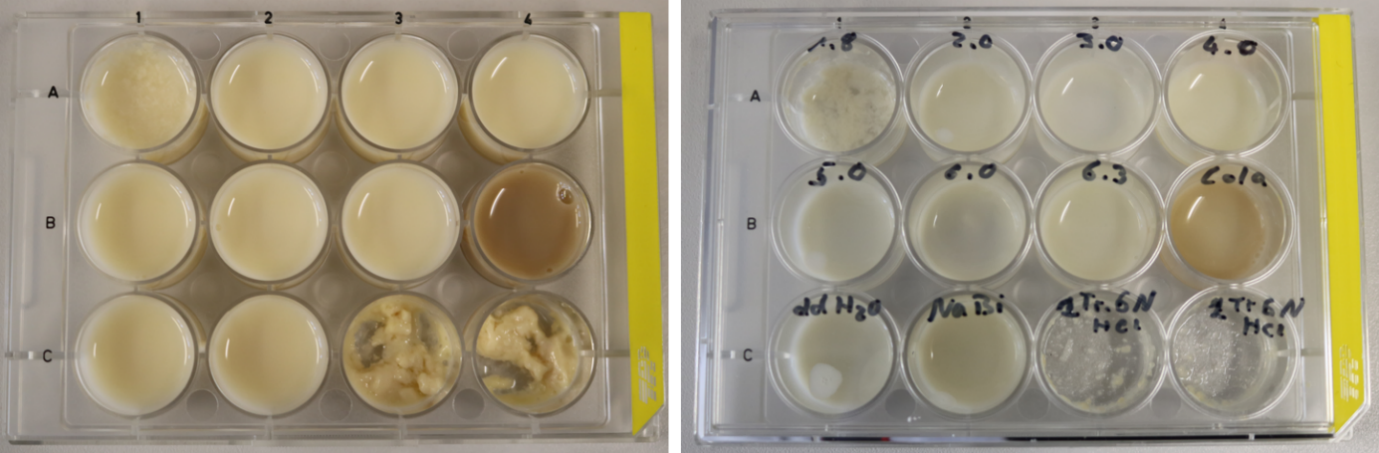


**Image 3:** In vitro solidification experiment. Left: PFP mixed with differing NaCl/HCL solutions, resulting in varying pH-values (see table 2). Right: Same wells after removal of supernatant. Wells A1, C3 and C4 showed solidification of PFP, whereas all other wells did not solidify.

| **Table 2:** In vitro solidification experiment - pH-values | | | | | |
| --- | --- | --- | --- | --- | --- |
|  |  | **1** | **2** | **3** | **4** |
| **A** |  | **NaCl/HCL, pH 1.8** | **NaCl/HCL, pH 2.0** | **NaCl/HCL, pH 3.0** | **NaCl/HCL, pH 4.0** |
|  | **pH-value** | 4.5 | 6.5 | 6.2 | 6.2 |
|  |  |  |  |  |  |
| **B** |  | **NaCl/HCL, pH 5.0** | **NaCl/HCL, pH 6.0** | **NaCl/HCL, pH 6.3** | **Coca-Cola ®** |
|  | **pH-value** | 6.2 | 6.2 | 6.3 | 5.8 |
|  |  |  |  |  |  |
| **C** |  | **ddH20** | **NaHCO_3_ 8.4%** | **6N HCL** | **6N HCL** |
|  | **pH-value** | 6.6 | 8.5 | Could not be measured, due to immediate clotting | Could not be measured, due to immediate clotting |


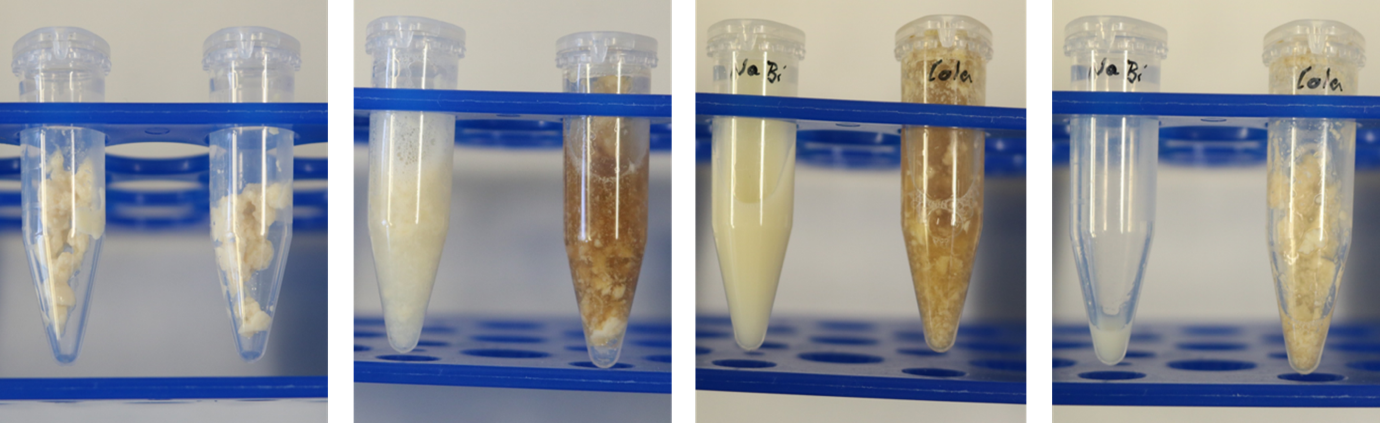


**Image 4:** Dissolution of clotted PFP; left tube filled with NaHCO_3_ 8.4%, right filled with Coca-Cola ®. Reactions immediately (B), at 30min (C), and after 3 hours after removal of supernatant (D). (B) left tube with immediate formation of bubbles; right tube without reaction; (C) left tube completely dissolved PFP, right tube with: persisting clots; (D) left tube no clots, right tube clotted PFP (no change to A).

**In vitro experiment of solidification and dissolution of Nepro HP ®**

In accordance to the above described experimental set up, Nepro HP ® was mixed with pure HCl, which led to an immediate solidification of the solution. In correspondence with the results shown above, adding NaHCO_3_ 8.4% led to a quick and complete dissolution of the artificially created bezoar. Image 5 illustrate the Nepro HP ® in vitro solidification and dissolution process.


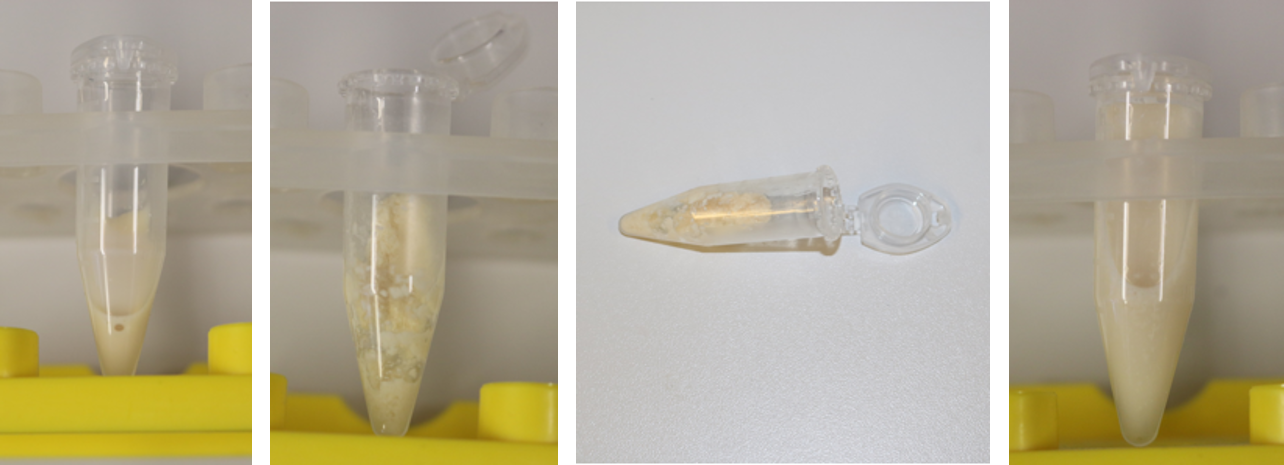


**Image 5:** In vitro solidification/dissolution experiment with Nepro HP ®. A: Nepro HP ® before adding HCl. B and C: Solidified Nepro HP ® after adding HCl and removal of supernatant. D: Complete dissolution of clotted Nepro HP ® after adding NaHCO_3_ 8.4% (image taken after 5 minutes).
